# Single Origin of Sex Chromosomes and Multiple Origins of B Chromosomes in Fish Genus *Characidium*

**DOI:** 10.1371/journal.pone.0107169

**Published:** 2014-09-16

**Authors:** José Carlos Pansonato-Alves, Érica Alves Serrano, Ricardo Utsunomia, Juan Pedro M. Camacho, Guilherme José da Costa Silva, Marcelo Ricardo Vicari, Roberto Ferreira Artoni, Cláudio Oliveira, Fausto Foresti

**Affiliations:** 1 Universidade Estadual Paulista (UNESP), Instituto de Biociências/IB, Departamento de Morfologia, Botucatu, São Paulo, Brazil; 2 Universidad de Granada, Departamento de Genetica, Granada, Spain; 3 Universidade Estadual de Ponta Grossa, Departamento de Biologia Estrutural, Molecular e Genética, Ponta Grossa, Paraná, Brazil; University of Florence, Italy

## Abstract

Chromosome painting with DNA probes obtained from supernumerary (B) and sex chromosomes in three species of fish genus *Characidium* (*C*. *gomesi*, *C. pterostictum* and *C. oiticicai*) showed a close resemblance in repetitive DNA content between B and sex chromosomes in *C. gomesi* and *C. pterostictum*. This suggests an intraspecific origin for B chromosomes in these two species, probably deriving from sex chromosomes. In *C. oiticicai*, however, a DNA probe obtained from its B chromosome hybridized with the B but not with the A chromosomes, suggesting that the B chromosome in this species could have arisen interspecifically, although this hypothesis needs further investigation. A molecular phylogenetic analysis performed on nine *Characidium* species, with two mtDNA genes, showed that the presence of heteromorphic sex chromosomes in these species is a derived condition, and that their origin could have been unique, a conclusion also supported by interspecific chromosome painting with a CgW probe derived from the W chromosome in *C. gomesi*. Summing up, our results indicate that whereas heteromorphic sex chromosomes in the genus *Characidium* appear to have had a common and unique origin, B chromosomes may have had independent origins in different species. Our results also show that molecular phylogenetic analysis is an excellent complement for cytogenetic studies by unveiling the direction of evolutionary chromosome changes.

## Introduction

Supernumerary (B) chromosomes are extra elements found in many eukaryotic genomes in addition to the standard (A) complement of chromosomes. They usually consist of highly repetitive DNA sequences, such as ribosomal DNA, satellite DNA, and mobile elements [1], [2], [3], while examples of B chromosomes carrying coding sequences [4], [5], [6], [7], functional ribosomal DNA [8], [9], and influencing sex determination [10] have recently been reported. In some cases, DNA sequences in the B chromosomes resemble those in the A chromosomes of the same genome (intraspecific origin) whereas, in others, they are more similar to DNA sequences in the genome of a relative species (interspecific origin) [11]. B chromosomes of intraspecific origin may be derived either from autosomes, as it is the case in *Locusta migratoria* [12], or from sex chromosomes, as in the frog *Leiopelma hochstetteri* [13], the fish *Lithocromis rubripinnis* [10] and the fly *Drosophila albomicans* [14]. In fact, sex and B chromosomes may share some features related to the distribution of chromatin, the accumulation of repetitive DNA and the loss of gene activity [11].

The presence of B chromosomes has been reported in several fish species [15], [16], [17], [10], [18]. Morphologically, these chromosomes are highly variable and range in size from very small, as in *Moenkhausia sanctafilomenae* [19] and *Prochilodus scrofa* [20], to very large, as in *Astyanax scabripinnis* [21], [22] and *Alburnus alburnus* [15]. In some cases, the repetitive sequences present on B chromosomes are shared with autosomal chromosomes, as in *P*. *lineatus* [23], [24] and *A*. *scabripinnis* [25], [26]. The B chromosomes of other species such as *A*. *alburnus* contain unique repetitive sequences [15].

Among Neotropical fish, the genus *Characidium* (of the family Crenuchidae) provides an interesting model for cytogenetic and evolutionary studies, particularly because of the presence of differentiated sex chromosome systems [27] and supernumerary chromosomes [28], [29], [30]. Despite the conserved diploid number of 50 chromosomes in all species in this group [29], interspecific and interpopulational differences have been reported in relation to either sex chromosomes of the ZZ-ZW type [31], [32], the location and number of rDNA sites [27], the occurrence of natural triploidy [33], [34] and the presence of B chromosomes [28], [29], [30].

In *Characidium*, it has been postulated that the sex chromosome systems found in several species have a common origin and that the observed differences between the Z and W chromosomes in different species and populations were caused by a variety of structural rearrangements, mainly involving rDNA sites and heterochromatic regions [31], [32]. However, though most species show a single rDNA-carrying chromosome pair, its chromosome location also shows variation [31]. Some species and populations are particularly interesting because they are highly differentiated: (*i*) the population of *C*. *gomesi* at Pardo River shows cells with up to four B chromosomes and lacks rDNA in the sex chromosomes [35]; (*ii*) the population of *C*. *gomesi* from the Tietê River has rDNA on the Z and W chromosomes and does not have any B chromosomes [30]; (*iii*) the population of *C*. *oiticicai* from the Paraitinga River has cells with up to 3 small extra chromosomes and shows NORs on the long arms of the W chromosome and on the short arms of the Z chromosome [29]; and (*iv*) a population of *C*. *pterostictum* from the Betari River exhibits cells with up to 3 small B chromosomes, NORs on the long arms of the W chromosome and on the short arms of the Z chromosome, and an acrocentric pair that is exclusive of this species [29]. Here we analyse the origin of B chromosomes in these three *Characidium* species by means of chromosome painting with DNA probes obtained from the B and W chromosomes, and infer the direction of chromosome changes by a mitochondrial DNA phylogeny built with these three *Characidium* species and six close relatives. The results have shown a unique origin for the sex chromosome systems in this genus, but multiple origins for B chromosomes.

## Materials and Methods

### Ethics Statement

Samples were collected in accordance with Brazilian environmental protection legislation (collection permission MMA/IBAMA/SISBIO - number 3245), and the procedures for sampling, maintenance and analysis of the samples were performed in compliance with the Brazilian College of Animal Experimentation (COBEA) and was approved (protocol 595) by the BIOSCIENCE INSTITUTE/UNESP ETHICS COMMITTEE ON USE OF ANIMALS (CEUA).

### Origin of samples and cytogenetic analysis

**Figure 1:**
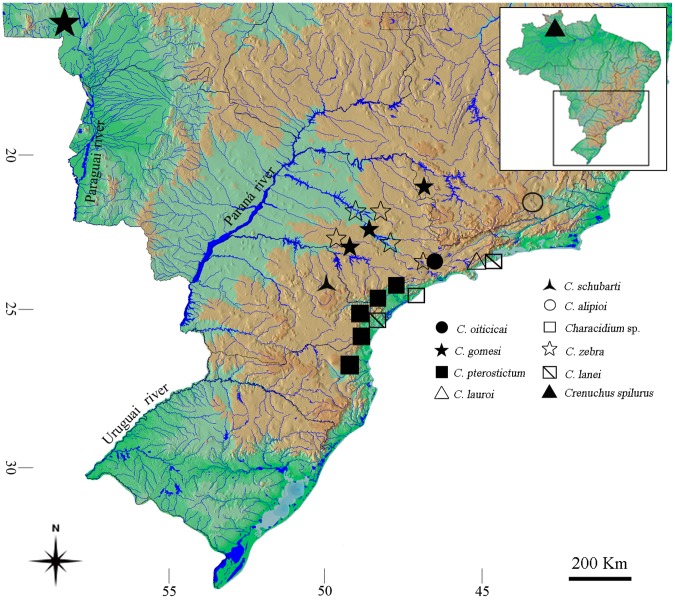
Map showing collection localities for each *Characidium* species.

For the phylogenetic analysis, we assessed a population of *Crenuchus spilurus* as an outgroup and the ingroup was composed by *C*. *zebra*, *C*. *gomesi*, *C*. *alipioi*, *C*. *lauroi*, *C*. *oiticicai*, *C*. *pterostictum*, *C*. *schubarti*, *C*. *lanei* and *Characidium* sp. (fig. 1). The animals were collected in accordance with Brazilian environmental laws (collection permission from MMA/IBAMA/SISBIO, number 3245). The collection, maintenance and analysis of the animals were performed in accordance with the international regulations on animal experimentation followed by the University of the State of São Paulo (CEEAA/IBB/UNESP Protocol number 304). For the cytogenetic analysis, the animals were anesthetised and dissected, and the mitotic chromosomes were obtained from renal tissue and gills using the technique described by Foresti et al. [36]. C-banding was performed following the protocol described by Sumner [37]. The samples to be analysed were identified, fixed and deposited in the fish collection of the *Laboratório de Biologia e Genética de Peixes*, Botucatu, São Paulo, Brazil (table 1).

**Table 1:** *Characidium* species and populations analyzed.

							A chromosomes				
Species	LPB	Location	Geographical Coordinates		F	M	m	sm	a	rDNA	Bs
*C. gomesi*	6723	Tietê River – Botucatu –SP	22°56′06 S	48°19′18 W	22	8	32	18	-	ZW	-
*C. gomesi*	6733	Novo River – Avaré – SP	23°01′26 S	48°49′32 W	9	20	32	18	-	17	0-4
*C. gomesi*	9021	São Domingos River – Muzambinho – MG	21°20′47 S	46°28′08 W	6	4	32	18	-	17	-
*C. gomesi*	9019	Vermelho River – Tangará da Serra – MT	14°35′25 S	57°42′35 W	8	5	32	18	-	17	-
					45	37					
*C. pterostictum*	7367	Betari River – Apiaí – SP	24°33′73 S	48°40′16 W	11	13	32	16	2	ZW	0-3
*C. pterostictum*	6831	Faú River – Miracatu – SP	24°12′44 S	47°28′61 W	6	2	32	16	2	ZW	-
*C. pterostictum*	768	Cari River – Morretes – PR	25°29′19 S	48°49′97 W	8	4	32	16	2	ZW	-
*C. pterostictum*	8701	Jacareí River – Paranaguá – PR	25°32′23 S	S48°4′19 W	8	3	32	16	2	ZW	-
*C. pterostictum*	737	Itapocu River – Jaraguá do Sul – SC	26°28′25 S	49°10′95 W	4	1	32	16	2	ZW	-
					26	10					
*C. oiticicai*	8730	Paraitinguinha River – Salesópolis – SP	23°30′40 S	45°51′32 W	8	6	32	18	-	ZW	0–3

LBP: Fish Collection of the Biology and Genetics Laboratory of Fish at Botucatu. F: females, M: males, m: metacentric, sm: submetacentric, a: acrocentric. rDNA: Chromosome pair carrying the 18S ribosomal DNA.Bs: B chromosomes.

### Microdissection and FISH

**Figure 2:**
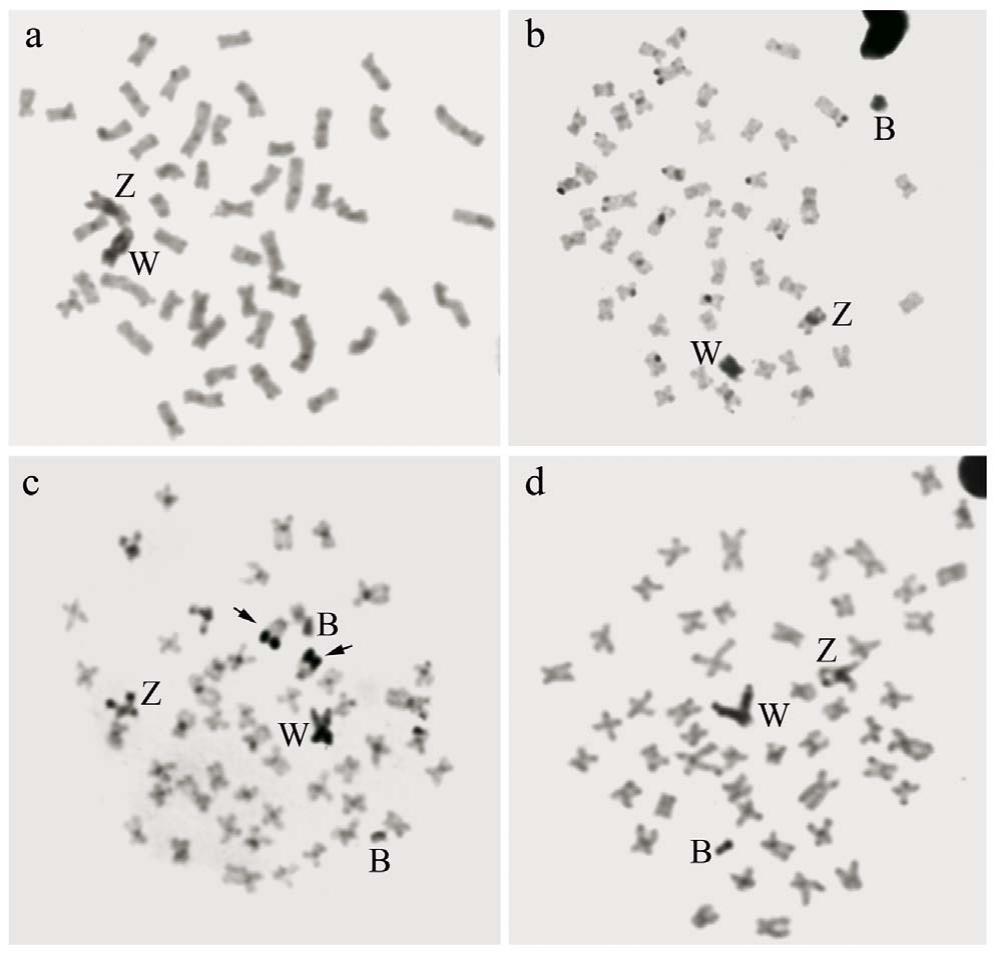
Metaphase chromosome spreads after C-banding. a) *C.* *gomesi* (Alambari River), b) *C.* *gomesi* (Paranapanema River), c) *C.* *pterostictum* (Betari River), d) *C.* *oiticicai* (Paraitinguinha River). The scale bar equals 10 µm.

For the microdissection of B chromosomes, we utilised cell suspensions from *C*. *gomesi* (Pardo River, registration number 33637), *C*. *pterostictum* (Betari River - registration number 37903) and *C*. *oiticicai* (Tietê River, registration number 31212) whose metaphases possessed a single B chromosome. For the microdissection of the W chromosome, we used a cell suspension from *C*. *gomesi* (Tietê River - registration number 31099). The B chromosomes were easily identified because they are smaller than the other chromosomes of the standard complement (A chromosomes) and have a distinct morphology (fig. 2B–D). The W chromosome from the Tietê River *C. gomesi* population has a secondary constriction in its long arm (fig. 2A). The cell suspensions were dropped on 24 mm×60 mm glass coverslips and stained with 5% Giemsa for 5 minutes, and the microdissection was performed using a micromanipulator with a glass needle (Eppendorf-5171) coupled to an inverted microscope (Zeiss Axiovert 100). Ten B chromosomes from each population and 12 W chromosomes from *C. gomesi* (Tietê River) were microdissected. These chromosomes were placed in four different microtubes (0.2 mL) with 9 µL of ultrapure water, and the DNA was amplified using the GenomePlex Single Cell Whole Genome Amplification Kit (WGA4 - Sigma) [38]. The reaction products were visualised on agarose gels. We generated probes for the B chromosomes (CgB - *C. gomesi*, CpB - *C. pterostictum*, CoB - *C. oiticicai*) and for the W chromosome (CgW - *C. gomesi*) from a re-amplification reaction of the amplified DNA and the GenomePlex WGA Re-amplification Kit (WGA3 - Sigma) using the modified nucleotide digoxigenin-11-dUTP (Roche Applied Science).

Fluorescent *in situ* hybridisation (FISH) experiments using probes specific to the B chromosomes were performed on four individuals per species, including B-carrying and B-lacking males and females. The *in situ* hybridisation was performed as described by Pinkel et al. [39], under high stringency conditions (mix containing 200 ng of each probe, 50% formamide, 10% dextran sulphate, 2xSSC at 37°C overnight). The chromosomes were counterstained with 4′, 6-diamidino-2-phenylindole dihydrochloride (DAPI) and analysed using an optical photomicroscope (Olympus BX61). The images were captured using the Image Pro plus 6.0 software (Media Cybernetics) and processed using the Adobe Photoshop CS2 program to improve their brightness and contrast.

### Analysis of mitochondrial DNA

To extract DNA, the PromegaWizardGenomic DNA Purification Kit was used according to the manufacturer's instructions. We used partial Cytochrome oxidase C subunit1 (COI) gene and Cytochrome B gene sequences (Cyt B) for molecular phylogenetic analyses. For the amplification and sequencing of the gene segments, we used the primers FishF1 5′TCA ACC AAC CAC AAA GAC ATT GGC AC3′ and FishR1 5′TAG ACT TCT GGG TGG CCA AAG AAT CA 3′ [40] to COI and Cyt B L 14841 5′ CCA TCC AAC ATC TCA GCA TGA TGA AA 3′ and Cyt B H 15915b 5′ AAC CTC CGA TCT TCG GAT TAC AAG AC 3′ [41] to Cyt B.

The corresponding sequences were amplified by PCR in a total reaction volume of 12.5 µl with 35 cycles (240 s at 95°C, 45 s at 48–54°C, and 60 s at 72°C). For the PCR, we employed the Gotaq kit (Promega) according to the manufacturer's recommendations. The PCR products were analysed on 1% agarose gel and purified using ExoSAP-IT reagent (USB Corporation) according to the manufacturer's instructions. The purified products were then used to assemble sequencing reactions with the BigDye TM Terminator v3.1 Cycle Sequencing Ready Reaction Kit (Applied Biosystems) in accordance with the instructions provided in the user's manual. The reactions were purified, and the sequences were obtained using an ABI3130 Automated Capillary DNA Sequencer. The sequences were initially analysed using the ATGC program (Genetix Inc.) to obtain consensus sequences. The consensus sequences were aligned using the MUSCLE algorithm [42]. Each gene block was aligned separately and then concatenated into a single matrix. The matrix was partitioned into six partitions, one partition for each codon position of each gene. A matrix saturation test was conducted with the DAMBE program version 5.1.1 using the method described by Xia et al. [43] and in the same program by the transition/transversion rate graphic. The relationship between the sampled taxa using Maximum Likelihood (ML) analysis was performed using the ‘RAxML-HPC2onXSEDE’ tool [44]. ML analyses were conducted under GTR+G since RAxML only applies this model [44]. A bootstrap test with 1,000 pseudoreplicates [45] was used as a statistical test of the phylogeny, nodes with bootstrap values below 70% were collapsed. An independent Bayesian analysis was conducted. Two independent MCMC chains were run with 5,000,000 replicates each, sampling one tree every 1000 steps. The search for the best model of nucleotide evolution for each partition was performed using the model test 3.6 [46] using the Akaike information criterion [47]. The distribution of log likelihood scores was examined to determine stationarity for each search and to decide if extra runs were required to achieve convergence, using the program Tracer 1.4 [48]. Initial trees estimated prior to convergence were discarded as part of a burn-in procedure, and the remaining trees were used to construct a 70% majority rule consensus tree in Paup* [49].

### Statistical Analysis

We compared the prevalence of B chromosomes between males and females, in each species, by means of contingency chi square tests, with Yates' correction, using the GraphPad PRISM 5.1 software.

## Results

The standard karyotype of all *Characidium* species hitherto analysed consists of 50 standard (A) chromosomes. In addition, the three species analyzed here (*C. gomesi* - Pardo River, *C. oiticicai*- Paraitinguinha River and *C. Pterostictum*- Betari River) harbour mitotically unstable B chromosomes, i.e. varying in number among the cells within a single individual. In all three species, the B chromosomes were the smallest members of the karyotype. *C*. *gomesi* and *C*. *oiticicai* showed very similar size groups for the A chromosomes, with 32 metacentric (m) and 18 submetacentric (sm) chromosomes, whereas *C*. *pterostictum* showed 32 m, 16 sm and 2 acrocentric (a) chromosomes. All three species showed a ZZ/ZW sex chromosome system (Table 1), similar to most other *Characidium* species hitherto analysed.

Only one of the four *C. gomesi* populations analyzed, collected in the Pardo River (Table 1) showed B chromosomes, with 72% of individuals carrying them (table 2). Likewise, only one of the five *C*. *pterostictum* populations analyzed, from the Betari River, carried B chromosomes (62% of B-carriers) (table 2). Finally, 50% of the individuals analyzed from the single *C*. *oiticicai* population sampled (Paraitinga River) carried B chromosomes (table 2). Contingency chi square tests failed to show significant differences in B chromosome prevalence between females and males, in any of the three species (table 2).

**Table 2:** Proportion of individuals carrying B chromosomes (prevalence).

		B^−^	B^+^	Total	Prevalence	?^2^	df	P
*C. gomesi*	Females	7	12	19	0.632	1.21	1	0.271
	Males	1	9	10	0.900			
	Total	8	21	29	0.724			
								
*C. pterostictum*	Females	3	10	13	0.769	1.35	1	0.245
	Males	6	5	11	0.455			
	Total	9	15	24	0.625			
								
*C. oiticicai*	Females	3	3	6	0.500	0.29	1	0.589
	Males	4	4	8	0.500			
	Total	7	7	14	0.500			

Proportion of individuals carrying B chromosomes (prevalence) in three species of fish genus *Characidium*. The B-carrying population in each species is indicated in Table 1. χ^2^ =  Yates's corrected contingency chi square test comparing the frequency of B-carrying (B^+^) and B-lacking (B^−^) females and males.

The C-banding technique revealed very similar patterns in the three species, especially for the W and B chromosomes, which were completely darkly C-banded in all three species (fig. 2). The Z chromosome showed a large pericentromeric C-band in all analysed populations of *C*. *gomesi* and *C*. *oiticicai*, but it showed an additional distal C-band in *C*. *pterostictum*. The autosomes showed pericentromeric C-bands in all three species. In *C*. *pterostictum*, there were also distal C-bands in the short arm of the submetacentric pair no. 22 and the long arm of the acrocentric pair no. 25.

**Figure 3:**
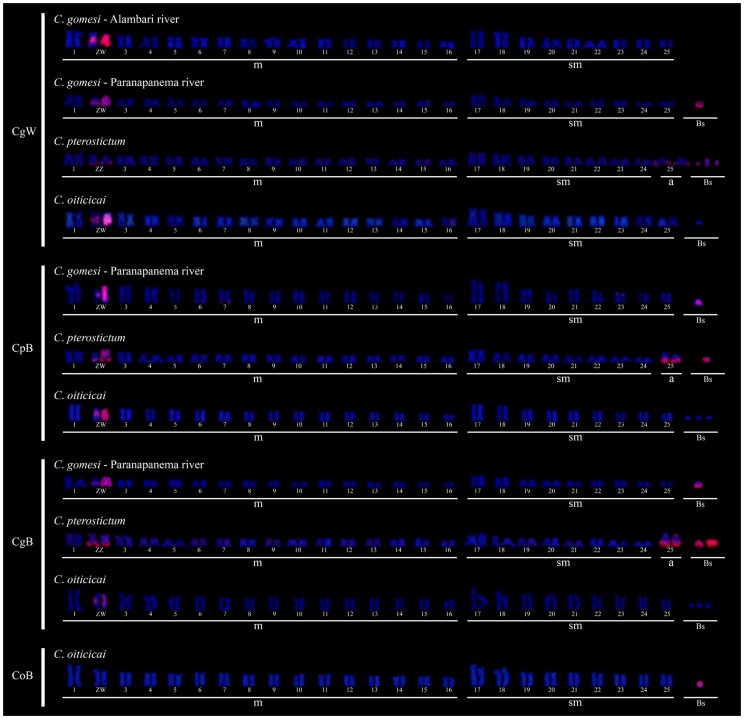
Karyotypes of *Characidium* species after chromosome painting with the CgW, CpB, CgB and CoB probes and counterstained with DAPI. m =  Metacentric; sm =  Submetacentric; a =  Acrocentric.

Intraspecific chromosome painting with the probes obtained from B chromosome microdissection (table 3) showed remarkable differences between the species. In *C*. *gomesi*, the CgB probe hybridised with the B chromosomes, the pericentromeric region of the Z chromosome, and the entire W chromosome (fig. 3). Likewise, in *C*. *pterostictum*, the CpB probe hybridised with the B chromosomes, the distal region of the Z chromosome long arm, the complete W chromosome and the distal region of the acrocentric pair no. 25 (fig. 3). In *C*. *oiticicai*, however, the CoB probe only hybridised with the B chromosomes (fig. 3). Hybridization signals near the telomere of some A chromosomes without a defined pattern have been observed in experiments with B chromosome probes for *C. gomesi* and *C. oiticicai*, presumably due to the presence, in the B-probes, of some repetitive DNAs being also present in some A chromosomes.

**Table 3:** Summary of *in situ* hybridisation experiments with the B and W chromosome probes.

		Painting probe			
Species	Chromosome	CgB	CpB	CoB	CgW
*C*. *gomesi*	B	+	+	-	+
	Z	+	+	-	+
	W	+	+	-	+
	A	-	-	-	-
					
*C*. *pterostictum*	B	+	+	-	+
	Z	+	+	-	+
	W	+	+	-	+
	A	+	+	-	+
					
*C*. *oiticicai*	B	-	-	+	-
	Z	+	+	-	+
	W	+	+	-	+
	A	-	-	-	-

Interspecific chromosome painting (table 3) with the CgB probe showed hybridisation with the B, Z, and W chromosomes and the acrocentric autosome pair no. 25 in *C*. *pterostictum*, but only with the sex chromosomes in *C*. *oiticicai* (fig. 3). Likewise, the CpB probe showed hybridisation signals on the sex and B chromosomes in *C*. *gomesi*, but only on the sex chromosomes in *C*. *oiticicai* (fig. 3). In high contrast, however, the CoB probe did not show any hybridisation signal on the *C*. *gomesi* and *C*. *pterostictum* chromosomes (table 3). Finally, the experiments using the CgW probe yielded the same results as those obtained with the CgB and CpB probes (fig. 3).

**Figure 4:**
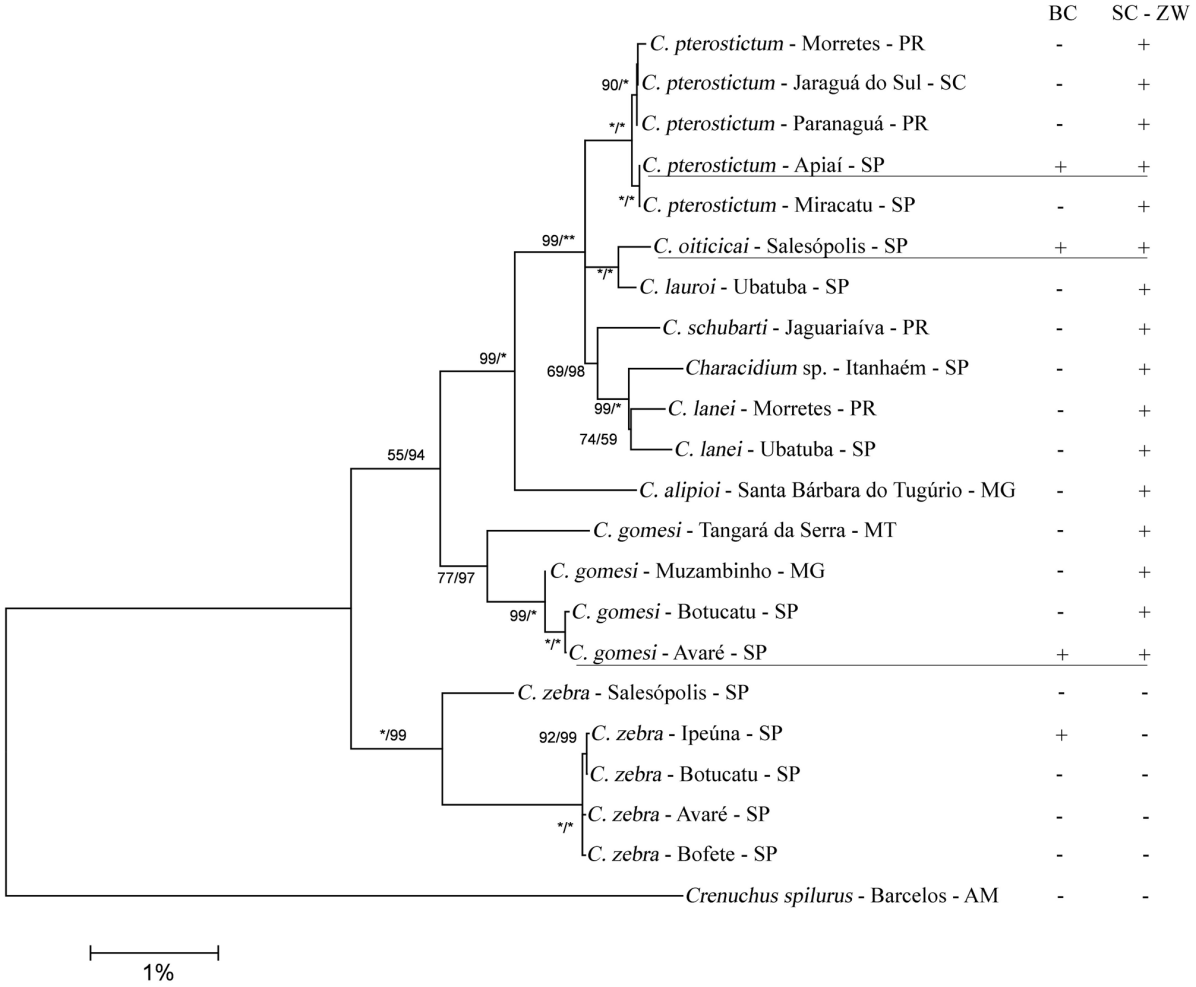
Consensus topology obtained by maximum likelihood and Bayesian analysis of the concatenated mtDNA dataset. The two numbers over each node represent the percentage of bootstrap obtained by ML and the posterior probability for that split obtained in the Bayesian analysis, respectively. When one of these indices is equal to 100, it is replaced by an asterisk.

To infer the evolutionary direction of the observed chromosome changes, we build a molecular phylogeny for mitochondrial DNA using the Cyt B (GenBank acession numbers from KF914671 to KF914692) and COI (GenBank accession numbers from KF914693 to KF914710) genes on nine *Characidium* species and an outgroup. We analysed a total of 1,694 nucleotides, 610 of which were variable, and 458 were parsimoniously informative. There was no saturation since the Iss.c value was greater than the Iss value, and the information found in this matrix was used in the subsequent phylogenetic analysis [43], [50]. The graphic analysis of transition and transversion vs. genetic distance implemented by DAMBE 5.2.31 also indicated that the data were not saturated (R^2^ = 0.79 for transition; R^2^ = 0.90 for transversion). The best nucleotide substitution models selected for each partition after model test analysis are shown in Table S1. The topologies yielded by ML and Bayesian analyses were very similar, with high statistical support and posterior probability. The final topology, resulting from the strict consensus between the topologies of both analyses (fig. 4), showed the existence of two main clades, a basal one including all populations of *C*. *zebra* (a species lacking sex chromosomes), and the other clade including the remaining species (all of them carrying sex chromosomes). This finding strongly suggests that the origin of sex chromosomes in these species was unique, and that their presence is a derived state. In addition, the tree in fig. 4 shows that *C*. *gomesi* belongs to a clade that is rather distant from the one including *C*. *pterostictum* and *C*. *oiticicai*. This result indicates that the B chromosomes in *C*. *gomesi* and *C*. *pterostictum* presumably showed independent origins despite the high resemblance in their DNA content suggested by chromosome painting.

## Discussion

### Sex chromosome evolution

The molecular phylogenetic analysis performed on the mtDNA of nine *Characidium* species revealed the existence of two main clades, with *C. zebra* (a species lacking sex chromosomes) constituting the basal clade, and a derived clade including eight species showing wide geographical distribution [27], [51], [29], [31], [32] and carrying ZW♀/ZZ♂ sex chromosomes (see fig. 4). This suggests a unique origin for sex chromosomes in this genus, and this conclusion is reinforced by the fact that the CgW probe hybridized with the sex chromosomes of the three species analyzed here. The existence of the same repetitive DNAs in the Z and W chromosomes of, at least, the three *Characidium* species analyzed, thus suggests the possibility that these sex chromosomes arose in an ancestor species. A single origin has also been suggested in fish genus *Triportheus* [52], but other genera of Neotropical fish, such as *Eigenmannia*, exhibit the opposite situation, with different sex chromosome systems being observed in closely related species, with presumable different and recent origins [53]. The joint cytogenetic and phylogenetic analysis of more representatives of *Characidium*, and related genera, also including DNA probes obtained from the Z chromosome, would provide a more general picture on sex chromosome evolution in this group of species.

### B chromosome evolution

The presence of B chromosomes in the genus *Characidium* appears to be limited to only a few populations in several species. Out of nine species with known cytogenetic data, B chromosomes have been found in a single population of *C*. *gomesi* (out of nine populations analysed), *C*. *pterostictum* (out of five populations analysed), *C*. *zebra* (out of seven populations analysed) and *C*. *oiticicai* (only one population analysed) [28], [33], [54], [55], [29], [30], [31]. The B chromosomes observed in these species were all mitotically unstable, but exhibited some differences regarding shape and heterochromatin distribution [28], [30], as expected for genomic elements evolving in separate species.

The DNA probes obtained from the B chromosomes of *C*. *gomesi* and *C*. *pterostictum*, and the W chromosome of *C*. *gomesi*, showed the same hybridisation pattern on the B, W and Z chromosomes of these two species, indicating that similar types of repetitive DNA make up their B and sex chromosomes. This probably reflects a descent relationship between these chromosomes and thus an intraspecific origin for these B chromosomes. As our molecular phylogenetic analysis has clearly shown, sex chromosomes were present in eight species constituting a well-defined clade, whereas B chromosomes were present in a single population of only three of these species. Therefore, sex chromosome origin preceded B chromosome origin, for which reason we can infer that the B chromosomes sharing DNA sequences with sex chromosomes in *C*. *gomesi* and *C*. *pterostictum* probably derived from sex chromosomes in each species. A possible caveat to this conclusion could be due to the fact that the DNA probes obtained by the GenomePlex method appear to contain a predominance of A+T rich DNA [56], and most painting signals observed by us were located on DAPI^+^ (i.e. A+T rich) regions. In fact, the probe obtained from the W chromosome (which contains rDNA, a kind of G+C rich DNA sequences) in *C. gomesi* (CgW) showed no hybridisation signals on the distal region of the autosome pair no. 17 (also carrying rDNA) of *C*. *gomesi* from the Pardo River population (fig. 3), suggesting that the probe did not contain rDNA sequences. But, in spite of this bias, it is clear that the repetitive DNAs included in the probes suggested a high resemblance in DNA content between B and sex chromosomes in *C. gomesi* and *C. pterostictum*, and thus these Bs could have conceivably arisen from sex chromosomes. Similar derivation from sex chromosomes has been shown in the frog *Leiopelma hochstetteri* [13]. Similarity of DNA sequences between B and A chromosomes, thus suggesting the intraspecific origin of B chromosomes, has also been reported in the fish species *Astyanax scabripinnis* [25], [26], *Prochilodus lineatus* [23] and *Lithocromis rubripinnis* [10], the grasshoppers *Eyprepocnemis plorans* [57], [58], [59] *Podisma sapporensis* [60] and *Locusta migratoria* [12] and, the mouse *Apodemus peninsulae* [61].

Alternatively, B chromosomes in these species could have derived from autosomes and later acquired the repetitive elements they share with sex chromosomes, but this event should have occurred in the two species separately, given their phylogenetic distance (see Fig. 4) and is thus unlikely.

In *C*. *pterostictum*, the CpB, CgB and CgW probes showed also hybridization on the heterochromatic blocks of the autosomal acrocentric pair no. 25. Therefore, in this species, B chromosomes could have also derived from this A chromosome. Nevertheless, the similarity of DNA sequences between the acrocentric pair and sex chromosomes in populations lacking B chromosomes, such as Miracatu, Morretes, Paranaguá and Jaraguá do Sul (results not shown) indicates that these shared sequences had settled in the acrocentric pair prior to B origin in the Betari River population. Therefore, given the highly dynamic nature of repetitive DNA sequences and their ability to spread across chromosomes within a same genome, the former conclusions should be taken with caution since chromosome painting results, based only in sequence similarity, could in fact be supporting a false idea of common descent between sex and B chromosomes. Comparison of nucleotide sequence will provide more precise conclusions at this respect.

Our molecular phylogenetic analysis suggests that the B chromosomes in *C. gomesi* and *C. pterostictum* most likely had independent origins, as these two species belong to very different clades. The fact that B chromosomes in both species are found in only one population could point to a recent origin which has not yet been followed by spread to other populations. An estimation of gene flow among populations in these two species would throw much light on this hypothesis. Alternatively, the B chromosomes in these two species could have derived from a B chromosome already present in a common ancestor species. But this would require the loss of the ancestor B chromosome in some species and populations, which is hardly parsimonious.

In contrast to the B probes obtained in the two other species, that obtained in *C. oiticicai* failed to hybridize with any chromosome other than the B chromosome itself, The lack of hybridization with any A chromosome has already been observed in chromosome painting experiments for a human supernumerary microchromosome, indicating the absence of detectable homologous sequences in the normal chromosomes, thus being the only supernumerary marker chromosome whose ancestry could not be determined [62]. These authors suggested possible mechanisms to explain the origin of this extra chromosome, including neocentromeric activity and either a complex amplicon of different genomic regions or the amplification of a very small region. Of course, these kinds of events cannot be ruled out to explain the origin of the B chromosome in *C. oiticicai* but, in fish, an alternative possibility is the interspecific origin through hybrydization. This has been suggested in several cases, such as the fish *Poecilia formosa* [63] or the wasp *Nasonia vitripennis* [64], and experimentally shown by Perfectti and Werren [65] inthe wasp *Nasonia*. The B chromosomes found in *C*. *oiticicai* showed some cytological similarities with those found in C. *pterostictum*, such as very similar size and heterochromatin content. But our chromosome painting and molecular phylogenetic results show that they can be B chromosomes independently originated in each species through different evolutionary pathways: intraspecific origin in *C. pterostictum* but interspecific origin in *C*. *oiticicai*. In other organisms, e.g. wasps, functionally equivalent B chromosomes showed independent origins, as shown for the paternal sex ratio (PSR) chromosomes in *Nasonia vitripennis* and *Trichogramma kaykai* [64], [66]. Ascertaining the precise introgressive hybridisation event that gave rise to the B chromosome in *C. oiticicai* is not an easy task, but a comparative analysis of DNA sequences contained in this B with those contained in the A chromosomes of this and other relative species, could be helpful [12], [67]. Two of the most interesting species for this research are *C*. *zebra* (which lives in sympatry with *C*. *oiticicai*) and *C*. *lauroi*, which is its closest relative species (see fig. 4). However, we cannot rule out that the amplification method used here may have privileged specific repetitive DNA sequences of B chromosomes being absent in the autosomes, and thus other DNA sequences presumably shared between As and Bs would have gone unnoticed with the employed methods.

## Conclusions

Our present results indicate that whereas sex chromosomes in the genus *Characidium* appear to have had a common and unique origin, B chromosomes in *C*. *gomesi*, *C*. *pterostictum* and *C*. *oiticicai* may have had an independent origin in every species, in the two former most likely derived intraspecifically from sex chromosomes, whereas in *C*. *oiticicai* they most likely arose through interspecific hybridization.

## Supporting Information

Table S1 Nucleotide substitution models for each partition used in the phylogenetic analyses of each program.
(XLSX)
